# Fighting Autism with Fatty Acids: Maternal Omega-3 Shields the Developing Brain from VPA-Induced Behavioral and Neurochemical Damage

**DOI:** 10.3390/biology14081065

**Published:** 2025-08-16

**Authors:** Emre Adıgüzel, Nuh Mehmet Bozkurt, Gökhan Ünal, Napoleon Waszkiewicz

**Affiliations:** 1 Department of Nutrition and Dietetics, Faculty of Health Sciences, Karamanoğlu Mehmetbey University, 70100 Karaman, Turkey; 2 Department of Pharmacology, Faculty of Pharmacy, Erciyes University, 38280 Kayseri, Turkey; mehmetbozkurt@erciyes.edu.tr (N.M.B.); gokhanunal@erciyes.edu.tr (G.Ü.); 3 Department of Psychiatry, Faculty of Medicine, Medical University of Białystok, 15-272 Białystok, Poland; napoleon.waszkiewicz@umb.edu.pl

**Keywords:** autism, omega-3, neuroinflammation, GAD67, parvalbumin

## Abstract

**Background/Objectives**: Autism spectrum disorder is a psychological condition characterized by symptoms such as repetitive stereotypic behaviors and social interaction/communication difficulties. It is known that omega-3 deficiency during brain maturation may cause learning disabilities and motor impairment. Therefore, we examined the effects of omega-3 treatment during gestation and/or lactation on autism-related behavioral and molecular deficits in a valproic acid (VPA)-rat model. **Methods**: Female Wistar rats were divided into five groups: control, VPA (500 mg/kg at G12.5), VPA+omega-3 (gestation), VPA+omega-3 (lactation), and VPA+omega-3 (gestation + lactation). The omega-3 supplement was dissolved in drinking water and offered for consumption daily during gestation and/or lactation. After the treatment period, behavioral tests were performed. The rats were then sacrificed, and inflammatory cytokines, parvalbumin, and glutamate decarboxylase-67 (GAD67) levels in the prefrontal cortex and hippocampus were examined. **Results**: Prenatal VPA administration increased repetitive behaviors, decreased sociability, impaired memory, and induced anhedonia. The behavioral and neurochemical effects of VPA exposure were more severe in males than in females. Early maternal omega-3 treatments rescued these behavioral changes. The treatments also reversed prenatal VPA-induced neuroinflammation. Lastly, GAD67 and parvalbumin decreases in these brain regions were mitigated by the treatments, the therapeutic effects of which were more pronounced in males. In terms of efficacy, the treatment groups ranked as follows: “gestation + lactation” > “gestation” > “lactation”. **Conclusions**: Maternal omega-3 supplementation—especially when administered throughout gestation and lactation—provides significant protection against behavioral and neurochemical deficits associated with prenatal VPA exposure. Early omega-3 intake may serve as a valuable complementary strategy in autism intervention.

## Simple Summary

Autism is a neurodevelopmental condition that affects many behaviors, such as social interaction and communication, and many families struggling with autism are looking for safe and practical ways to support healthy brain development. This study tested whether supplementing pregnant and lactating mothers with omega-3 fatty acids—a key component of brain cell membranes—can protect offspring in a rat model that shows autism-like features. A common anti-seizure medicine valproic acid was used to trigger these features in rats, after which we measured social interaction, memory, pleasure seeking, and repetitive grooming, as well as signs of inflammation in brain regions important for social skills and memory. Maternal omega-3 clearly helped: it reduced repetitive behaviors, restored social interaction and memory, and improved pleasure seeking. It also lowered brain inflammation and improved markers linked to calming nerve signals. Benefits were stronger in males, which showed more severe problems, and the best results appeared when omega-3 was given during both pregnancy and breastfeeding, compared with either period alone. These findings suggest that early omega-3 intake could be a low-risk, accessible addition to standard approaches aimed at reducing autism-related challenges, while careful human studies are still needed to determine the right timing, dose, and safety.

## 1. Introduction

According to the Diagnostic and Statistical Manual of Mental Disorders (DSM-5), autism spectrum disorder (ASD) is a psychological condition characterized by two core symptoms: repetitive/stereotypic behaviors and social interaction/communication impairment. Decreased nociception, anxiety, reduced exploratory behaviors, spatial working memory impairments, anhedonia, and depression are also common in individuals with autism [1,2,3,4].

There are no clear anatomical, pathological, or biochemical hallmarks of the underlying mechanism of autism [5]. However, changes in peripheral and neural proinflammatory cytokine, serotonin, gamma-aminobutyric acid (GABA) and glutamate levels are common molecular findings [6,7,8]. Postmortem brain tissue studies have shown increased activation of microglia and astroglia and thus increased proinflammatory cytokine levels in autism [9,10]. It is suggested that neuroinflammation interacts with environmental and genetic factors that contribute to autism, disrupting neuronal network organization and synaptic balance (notably the GABA/glutamate ratio) in early developmental stages. Thus, a chronic low-level neuroinflammatory process may underlie the social communication impairments and repetitive behaviors characteristic of autism [11,12]. Furthermore, neuropathological studies have revealed findings such as irregular neocortical architecture and clusters of misplaced neurons [13,14]. Other research has shown that synaptic pruning is disrupted and the glutamatergic dendritic spike density is increased [15], while lower sensorimotor GABA levels have been reported in autistic individuals compared to controls [16]. In fact, the suggestion that GABA, the main source of inhibitory transmission, is affected in autism was made following the discovery of decreased GABA receptor density [5]. Subsequently, changes in the density of GABAergic interneurons were found in the hippocampus and cerebellum of individuals with autism [17,18]. These discoveries led to the hypothesis of an altered excitation/inhibition (E/I) balance in autism. In the early twenty-first century, a model in which GABAergic inhibition is suppressed and the E/I balance shifts toward excitation was proposed as the basis for pathological brain circuit functioning in autism [19,20]. The most critical mechanism proposed for the shift in E/I imbalance toward excitation is reduced firing of parvalbumin-positive neurons, the largest class of GABAergic interneurons [5]. Parvalbumin is a Ca^2+^-binding protein. Action potentials in parvalbumin-positive neurons are characterized by high-frequency firing with minimal adaptation, a short membrane time constant, and long afterhyperpolarization [21]. There is increasing evidence in the literature that parvalbumin-positive neuron dysfunction is directly related to the pathogenesis of autism [22,23,24]. Other types of interneurons may also be involved in the pathology of autism but have received less research attention because *PVALB* is among the most down-regulated genes in the autistic cerebral cortex [5]. Nevertheless, the molecular mechanisms associated with GABA and GABAergic synaptic transmission underlying autism remain unclear [25].

It is of great importance to use models with high construct and face validity in experimental studies on autism. Some models that mimic the autism phenotype are well accepted, including the prenatal valproic acid (VPA) exposure model [26]. VPA exposure, especially in the second trimester, may induce the autism phenotype by affecting some neurodevelopmental mechanisms and pathways [27]. Prenatal VPA exposure is thought to alter E/I balance. It has been shown to cause significant histological changes in the prefrontal cortex, hippocampus, and cerebellum, such as decreased levels of parvalbumin-positive interneurons [28]. VPA also affects the activity of glutamate decarboxylase (GAD), which is involved in GABA synthesis [29], and can cause mitochondrial dysfunction that disrupts fatty acid metabolism and microbial colonization disturbances that can alter the short-chain fatty acid composition in the gastrointestinal system [30,31,32]. These abnormalities contribute to the pathogenesis of autism through energy metabolism disorders and immune/neuroinflammatory processes [33,34]. All these mechanistic effects suggest that the prenatal VPA exposure model has strong construct and face validity for autism [32].

Fatty acids are an essential component of cell membrane phospholipids with specific functional, metabolic, and signaling roles. In particular, omega-3 polyunsaturated fatty acids play a major role in maintaining membrane elasticity. The main essential omega-3 fatty acids are alpha-linolenic acid (ALA), eicosapentaenoic acid (EPA), and docosahexaenoic acid (DHA). These fatty acids have anti-inflammatory, antioxidant, antilipidemic, antithrombotic, and anti-diabetic effects. They also support mitochondrial β-oxidation [35,36]. In addition to their general health effects, these fatty acids are involved in activities vital for the nervous system, such as neurotransmission, neuroplasticity, and signal transduction [35]. They have attracted considerable attention in the field of fetal neurodevelopment due to their critical role in the growth and function of the developing brain. Adequate omega-3 intake during gestation and lactation promotes early brain development through peroxisome proliferator-activated receptor gamma (PPAR-γ) activation [37]. DHA in particular fights against neuro-oxidation and shows anti-neuroinflammatory activity. Membrane receptor function is critical for membrane fluidity and neuronal signaling and vice versa [38]. An omega-3 fatty acid-enriched diet was associated with the expression of some genes involved in synaptic plasticity and learning. It was found that glutamate receptor expression was altered in rodents fed an omega-3-deficient diet during the perinatal period [39]. It has also been reported that depriving the rat brain of omega-3 fatty acids during neurodevelopment disrupts plasticity [40]. Another study reported that consuming an omega-3-deficient diet for several generations resulted in impaired neuropeptide Y1 and glucocorticoid receptor expression in the hippocampus, leading to increased stress response and anxiety [41]. It is also important to note that omega-3 deficiency during brain maturation is associated with learning disabilities, motor impairments, and monoamine transmission dysfunction [38].

The accumulation of DHA, the main neuro-structural omega-3 fatty acid, in the brain accelerates during the third trimester (~14.5 mg/week). At term birth, DHA comprises approximately 9% of total cortical fatty acids, which increases to about 15% at age 20. In preterm infants (<33 weeks gestation), cortical DHA concentrations were reported to be approximately 40% lower than in full-term infants. These findings suggest that DHA largely accumulates in the human brain during the third trimester and continues to accumulate throughout brain maturation after birth [42]. Preterm children and adolescents have been reported to exhibit higher rates of attention deficits, impulsivity, learning disabilities, language disorders, hyperactivity, anxiety, motor disorders, and poor social behaviors than their full-term peers [43,44,45,46]. Maternal dietary intake of omega-3 fatty acids during lactation is also important, as it determines the DHA reserve in breast milk [47]. Studies have reported that neurocognitive problems, such as poor visual recognition memory, psychomotor impairments, problem-solving deficits, and lower intelligence quotient scores (IQs) are less common in infants and children who were breastfed or fed DHA-containing formula after birth than in those fed non-DHA-containing formula [48,49,50,51,52].

Although many studies have validated the critical role of omega-3 fatty acids in neurobiological mechanisms, conflicting results regarding their therapeutic effects have been reported in studies conducted on autistic individuals [53,54,55,56,57,58]. Little is known about the protective effects of early omega-3 supplementation against autistic symptoms. Therefore, this study was planned to evaluate the effects of omega-3 treatment during gestation and lactation on autism-related behavioral symptoms and some neurochemical parameters in a VPA-induced experimental model of autism.

## 2. Materials and Methods

### 2.1. Animals and Experimental Design

This study used female Wistar rats and their male and female offspring All animals were purchased from Erciyes University Experimental Research and Application Center (DEKAM) and all experimental procedures were performed at DEKAM. The animal-related procedures were approved by the Erciyes University Animal Experiments Ethics Committee (Decision number: 23/022; Date: 1 February 2023). The experiments complied with ARRIVE guidelines and were performed in accordance with the UK Animals (Scientific Procedures) Act, 1986 and related guidelines, and EU Directive 2010/63/EU for animal testing. The animals were housed under standard conditions (23 ± 1 °C room temperature, 55–60% humidity, and 12-h light/dark cycle). Ad libitum access to water and food was provided. The experiment room was illuminated between 07:00 a.m. and 07:00 p.m. during the experimental period. Ten female and ten male rats were caged at a male–female ratio of 1:1 for 12 h daily (07:00 p.m.–07:00 a.m.) until spermatozoa were observed in vaginal smears of the female rats. Vaginal smears were examined under a light microscope every morning at 08:00 a.m. The day on which spermatozoa were present was considered the first gestational day (gestational day 0.5 = G0.5). On this first gestational day (G0.5), each litter was assigned to either the control group or a treatment group. The most widely applied and accepted VPA dose to model autism is 500 mg/kg, which is administered on the 12.5th gestational day (G12.5) [59]. To establish the autism model in offspring assigned to a treatment group, eight pregnant rats were injected with a single dose of 500 mg/kg VPA (Sigma-Aldrich Chemical Co., St. Louis, MO, USA, Product no: P4543) intraperitoneally at G12.5. The VPA was dissolved in saline (dissolution volume 200 mg/mL). On the other hand, two pregnant rats whose offspring were assigned to the control group were injected intraperitoneally with the same volume of saline at G12.5. The pregnant rats were caged separately during gestation, and mother rats were caged with their pups during lactation. At the end of the gestation period, all rats gave birth to 6–12 pups. At least 2 of the pups from each dam were of different genders. To avoid variability in growth and development, some pups were culled on postnatal day 1 so that each dam was nursing a maximum of 8 pups. The animals were divided into 5 groups. Each group consisted of 2 mother rats and a total of 12 pups (6 males and 6 females), so that there were n = 6 pups per group for both males and females. The gestation period lasted 22–24 days. The lactation period lasted 21 days, and the pups were separated from their mothers on postnatal day 22 (P22). Seven days after the lactation period, the pups were subjected to behavioral tests (P28–P35). All rats were sacrificed, and brain tissues were dissected on P36.

The findings of Alfawaz et al. [60] were considered when determining the dose of omega-3. They reported that 30 days of omega-3 treatment at a dose of 200 mg/kg/day improved autism-like symptoms in rats. We aimed for an omega-3 intake close to these levels. Therefore, considering the water consumption of an adult rat (100–125 mL/kg), 425 mg (1/2 a capsule) of omega-3 was dissolved in a bottle of water (250 mL). Polysorbate-20 (ZAG Chemical Co., İstanbul, Turkey, Product no: ZS.100191.1000) was used for water solubilization of the omega-3 fatty acids. First, 1/2 an omega-3 supplement capsule was dissolved in 1 mL of polysorbate-20. This solution was then dissolved in water (water was added for a total volume of 250 mL). The groups that did not receive omega-3 treatment were given a polysorbate-20 solution containing 1 mL of polysorbate-20 and 249 mL of water. These solutions were prepared daily (at 07:00 p.m.) during the intervention period. The omega-3 fatty acid supplement (Solgar Inc., Leonia, NJ, USA) contained 53% EPA and 40% DHA.

The groups are described below.

*Control group (saline):* A single dose of saline was administered at G12.5. The mothers of pups in this group did not receive omega-3 treatment during gestation or lactation. The polysorbate-20 used as a solvent was administered during these periods.

*VPA (autism) group*: A single dose of VPA was administered at G12.5 to establish the autism model. The mothers of pups in this group did not receive maternal omega-3 treatment during gestation or lactation. The polysorbate-20 used as a solvent was administered during these periods.

*Gestation period omega-3 treatment group:* A single dose of VPA was administered at G12.5 to establish the autism model. The mothers of pups in this group received omega-3 treatment during gestation. In addition, the solvent polysorbate-20 was given during lactation.

*Lactation period omega-3 treatment group:* A single dose of VPA was administered at G12.5 to establish the autism model. The mothers of pups in this group received omega-3 treatment during lactation. The solvent polysorbate-20 was given during gestation.

*Gestation + lactation period omega-3 treatment group:* A single dose of VPA was administered at G12.5 to establish the autism model. The mothers of pups in this group received omega-3 treatment during both gestation and lactation.

**Figure 1:**
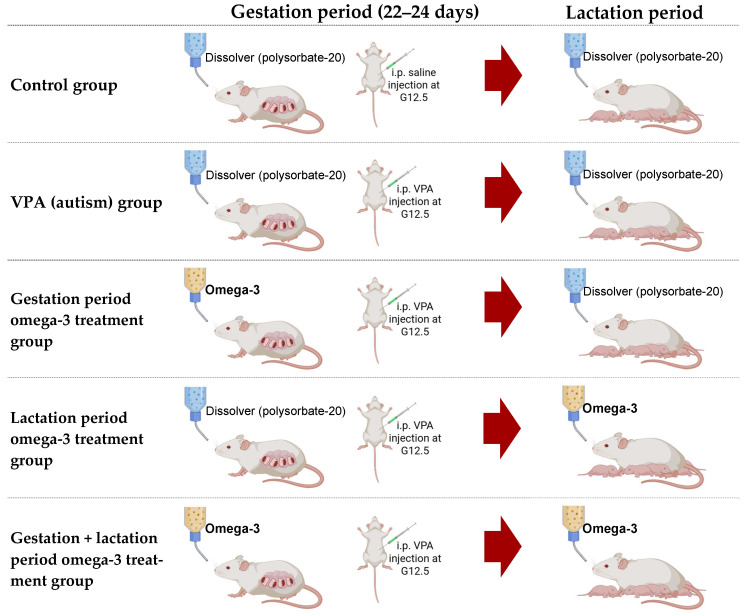
An overview of the intervention protocols during gestation and lactation. The treatment groups received omega-3 in one or both periods, while the control and VPA groups received only the dissolver (polysorbate-20).

The treatment schedule of each experimental group during gestation and lactation is presented in Figure 1.

**Figure 2:**
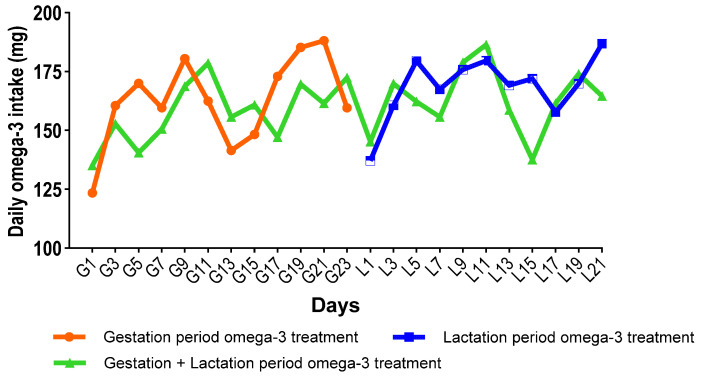
The daily average amount of omega-3 fatty acids consumed by each omega-3 treatment group. G: gestational day, L: lactational day.

The volume of solution consumed by the mothers in the omega-3 treatment groups was measured daily. The mean daily omega-3 intake during the treatment period was 162.7 ± 18.7 mg in the gestation period treatment group, 168.7 ± 13.4 mg in the lactation period treatment group, and 160.3 ± 13.7 mg in the gestation + lactation period treatment group (*p* > 0.05). Figure 2 shows the daily average amount of omega-3 fatty acids consumed in each omega-3 treatment group.

### 2.2. Behavioral Tests

Behavioral tests were started 7 days after the treatment period, and this phase lasted 7 days (P28–P35). To investigate the possible preventive effects of early omega-3 treatment on VPA-induced autism-like symptoms, we examined five important behavioral patterns that correspond to behavioral domains frequently altered in autism: stereotypy, sociability/social novelty, visual recognition memory, spatial memory, and anhedonia. These behaviors were assessed by repetitive self-grooming behavior (P28), three-chamber social interaction (P29–30), novel object recognition (P31), Y-maze (P32), and sucrose preference (P33–35) tests, respectively. All tests were performed between 10:00 a.m. and 04:00 p.m.

*Repetitive self-grooming behavior test:* Repetitive stereotypic behavior is one of the core symptoms of autism. In rodents, this phenotype manifests as behaviors such as self-grooming, digging, circling, and jumping. In this study, time spent grooming (face rubbing and paw/fur licking) in an open field environment was evaluated [61]. A 100 × 100 × 40 cm, open-ceiling, plexiglas apparatus was used as the test environment. The rats’ behavior in the test environment was recorded for 10 min using a video camera for 10 min. Grooming behavior was measured manually by a blinded scorer. Between trials, the inner surface of the apparatus was cleaned with 70% ethanol.

*Three-chamber social interaction test:* Another core symptom of autism is social behavior impairment. In rodents, this phenotype manifests as avoidant behavior when confronted with strange animals. The three-chamber social interaction test is usually preferred for the assessment of sociability. There are two main factors to consider when assessing the social behavior of rodents: (a) spending more time with a new conspecific than with an empty cage (sociability) and (b) spending more time with a new conspecific than with a familiar conspecific (social preference) [62]. The test protocol consists of two habituation phases (24 h before and immediately before sociability), a sociability task, and a social preference task [63]. On the first day, the first habituation phase was carried out, and on the following day, the second habituation phase and tasks were carried out. The test setup consisted of three open-ceiling chambers, each measuring 40 × 40 × 40 cm, placed side by side and connected by sliding doors. In the first habituation phase (15 min), the test animal was allowed to explore the setup. The second habituation phase (10 min) was for remembering the setup. In the sociability task, a stranger conspecific (stranger rat-1) was placed in the right chamber, an empty cage was placed in the left chamber, and the subject animal was placed in the middle chamber. The test started with opening the sliding doors, and the behavior of the subject animal was recorded by a video camera for 10 min. In the social preference task, the cages in the right and left chambers were switched, and a new stranger conspecific (stranger rat-2) was placed in the empty cage. The subject animal, which was moved back to the middle chamber after the sociability task, was not allowed to observe these procedures. After the setting was established, the sliding doors were opened again and the social preference task started. As in the sociability task, the behavior of the subject animal was recorded for 10 min. The time spent displaying attentive behaviors (climbing, pacing and sniffing) toward the stranger conspecifics or empty cage in the chambers was measured manually by a blinded scorer. Between trials, the cages and the inner surface of the apparatus were cleaned with 70% ethanol. The parameters examined in the sociability task were considered in the calculation of the “sociability index (SI)”, whereas the parameters examined in the social preference task were considered in the calculation of the “social preference index (SPI)”:

$$\mathrm{SI}=\frac{Time\;spent\;with\;stranger\;rat-1\;(s)}{Time\;spent\;with\;empty\;cage\;(s)}$$

$$\mathrm{SPI}=\frac{Time\;spent\;with\;stranger\;rat-2\;(s)}{Time\;spent\;with\;stranger\;rat-1\;(s)}$$

*Novel object recognition test:* While the three-chamber social interaction test evaluates social memory, the novel object recognition test evaluates object recognition memory. The test protocol reported by Bevins and Besheer [64] was applied in this study. A 50 × 50 × 40 cm open-ceiling plexiglass apparatus was used as the test environment. The test was carried out in 2 days, with the habituation phase on the first day and the test phase on the second day. In the habituation phase, the animals were allowed to explore the setup for 1 h. The test phase consisted of two trials, a “familiarization trial” and a “test trial” with a 1-h interval between them, and was carried out 24 h after the habituation phase. In the familiarization trial, two identical objects were placed in opposite corners of the setup (10 cm away from each wall). The subject animal was placed in the center of the setup and allowed to explore the objects for 3 min. In the test trial, which was carried out 1 h after the familiarization trial, one of the identical objects was replaced with a different object (novel object), and the subject animal was reintroduced the setup. In the test trial, the behavior of the subject animal was recorded by a video camera for 3 min. The time spent sniffing objects at a distance of less than 2 cm is considered the “exploration time”. In the test trial, exploratory behavior was measured manually by a blinded scorer. Between trials, the objects and the inner surface of the apparatus were cleaned with 70% ethanol. A discrimination index (DI) was calculated for each animal using the following formula:

$$\mathrm{DI}=\frac{Exploration\;time\;of\;novel\;object\;\left(s\right)-Exploration\;time\;of\;former\;object}{Exploration\;time\;of\;novel\;object\;\left(s\right)+Exploration\;time\;of\;former\;object}$$

*Y-maze test:* Animals tend to alternate between different options which requires the ability to remember the previous option. The Y-maze spontaneous alternation test has a simple protocol and is widely used to assess short-term memory in rodents. It considers the rodents’ behavior as they visit the arms of a Y-shaped open-ceiling setup. To visit the arms of the maze in the correct order, the subject animal needs to remember the arm it visited previously during the task [65]. The Y-maze test consists of habituation and test phases, which are conducted 24 h apart. In the habituation phase, the subject animal was placed in the setup and allowed to explore the arms for 5 min. In the test phase, the subject animal was placed in the setup again, and their arm visits were recorded by a video camera for 5 min [66]. Alternation is defined as sequential visits (such as ABC, BCA, CAB, ABC,...) to three different arms of the Y-maze. The inner surface of the setup was cleaned with 70% ethanol between trials. The spontaneous alternation rate was calculated for each animal using the following formula [67]:

$$\mathrm{Spontaneous}\mathrm{alternation}\;(\%)=\frac{Number\;of\;total\;alternations}{Number\;of\;total\;arm\;visits-2}\times 100$$

*Sucrose preference test:* The sucrose preference test is a reward-based behavioral test used to assess anhedonia. One of the underlying symptoms of depression is a decreased ability to experience pleasure. Rodents tend to consume sweet foods and drinks. A decrease in sweet preference represents anhedonia. The test protocol reported by Liu et al. [68] was applied in this study. First, two water bottles (both filled with water) were placed in the rats’ cages for three days prior to the start of the test phase to acclimatize the animals to the presence of two bottles in the cage. During these three days, the water decrease in each bottle was measured daily, and it was found that there was no preference for a specific bottle. In the test phase, the animals were caged individually, and each animal was offered two bottles, one containing 1% sucrose and the other containing only water, for 12 h. The test was carried out at night (between 07:00 p.m. and 07:00 a.m.) as circadian rhythm may influence consumption behavior. Subsequently, the animals were deprived of water and food for 24 h. Immediately after deprivation, each animal was offered two bottles again (1% sucrose and only water) for 12 h (07:00 p.m.–07:00 a.m.). To calculate the sucrose preference rate (%), the volumes of 1% sucrose solution and water consumed in the last 12 h were considered:

$$\mathrm{Sucrose}\mathrm{preference}\;(\%)=\frac{Sucrose\;solution}{Sucrose\;solution+Water}\times 100$$

### 2.3. Collection of Brain Tissue

A combination of ketamine (80 mg/kg) and xylazine (10 mg/kg) was used to anesthetize the rats before euthanasia. Each animal underwent cervical dislocation at the anesthetic depth at which the nociceptive flexion reflex disappeared. Euthanasia was followed by rapid removal of brain tissue and immediate dissection of the prefrontal cortex and ventral hippocampal tissues of the brain. The tissues were frozen on dry ice and stored at −80 °C until biochemical analysis.

### 2.4. Biochemical Analyses

Enzyme-linked immunosorbent assay (ELISA) was used to measure IL-1β, IL-6, TNF-α, interferon- γ (IFN-γ), glutamate decarboxylase-67 (GAD67), and parvalbumin levels in the prefrontal cortex and hippocampus. First, 50 mg of tissue was homogenized in 500 μL (0.1 M) of phosphate-buffered saline solution (PBS, pH: 7.4) for 5 min with an ultrasonic homogenizer. After the homogenized tissues were centrifuged at 10,000 rpm and 4 °C for 15 min, the supernatants were transferred to Eppendorf tubes and stored at −80 °C until the ELISA. The analyses were performed using a commercial ELISA kit (Shanghai Sunred Biological Technology Co., Shanghai, China, Product numbers: 201-11-0120 (IL-1β), 201-11-0136 (IL-6), 201-11-0765 (TNF-α), 201-11-0104 (IFN-γ), 201-11-2306 (GAD67), and 201-11-1444 (parvalbumin)), according to the manufacturer’s protocol. Total protein amounts in the samples were also determined to standardize the concentration values. A commercial bicinchoninic acid (BCA) protein assay kit (Thermo Fisher Scientific, USA) was used for this purpose. The concentration values determined by ELISA were divided by the total protein amounts to determine the concentration values per unit amount of protein.

**Figure 3:**
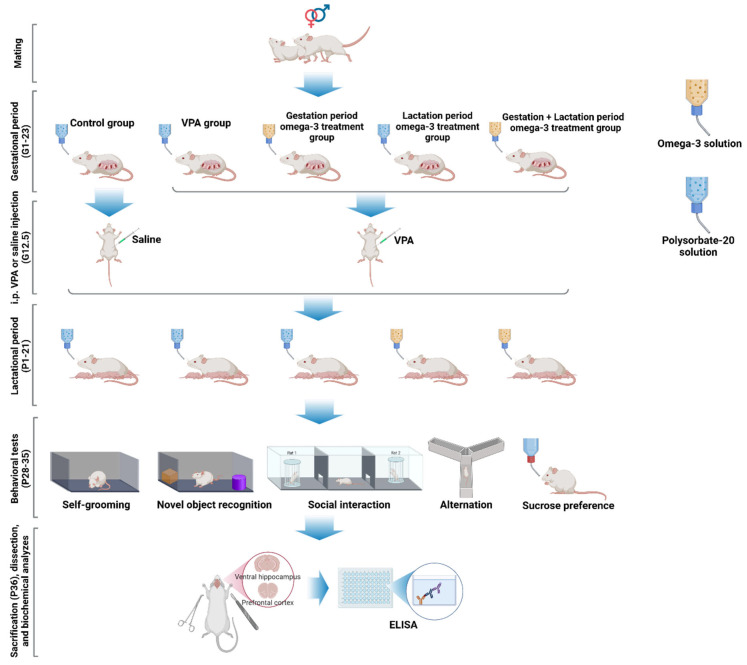
Overview of study methodology. After mating, pregnant Wistar rats (their litters) were randomly assigned to experimental groups and received either omega-3 fatty acids or solvent (polysorbate-20) orally during gestation and/or lactation. Each group consisted of 12 offspring (6 males and 6 females). Behavioral assessments included repetitive behavior (self-grooming), object memory (novel object recognition test), social interaction (three-chamber social interaction test), short-term memory (alternation), and anhedonia (sucrose preference test). Molecular analyses were performed on prefrontal cortex and hippocampus tissues to evaluate inflammatory markers (IL-1β, IL-6, TNF-α, and IFN-γ) and GABAergic system-related parameters (GAD67 and parvalbumin).

A methodological overview of this study is presented in Figure 3.

### 2.5. Statistical Analyses

The data are shown in graphs as “mean ± standard error of mean (SEM)” bars and individual triangular dots. Data normality was assessed using the Shapiro–Wilk test. Statistical differences between the groups were evaluated separately for each gender using one-way analysis of variance (ANOVA). Tukey’s multiple comparison test was selected for post–hoc evaluation. Comparisons were made based on VPA group, and statistical differences between all groups and the VPA group were considered. The statistical differences between genders were also evaluated separately for each group using Student’s *t* test. GraphPad Prism statistical graphics software (version 10.2.0; GraphPad Software Inc.; San Diego, CA, USA) was used for all analyses.

## 3. Results

**Figure 4:**
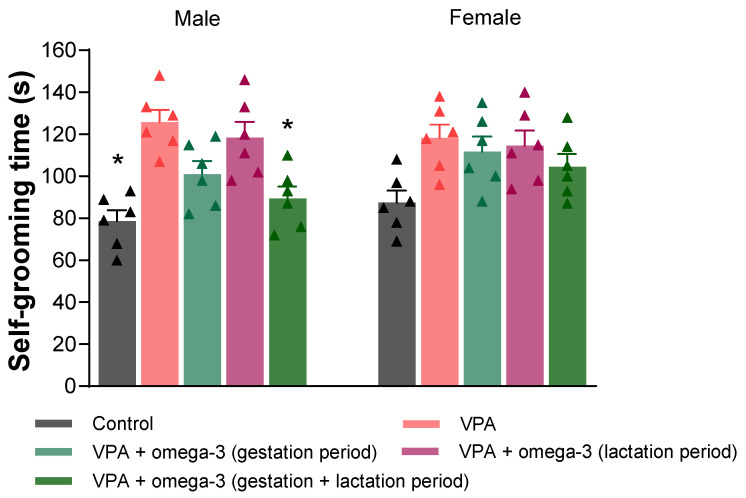
Duration of self-grooming behavior. Prenatal VPA administration increased the self-grooming time, and the gestation + lactation period omega-3 treatment reversed VPA-induced increase in self-grooming in male rats. There was no significant difference between the groups in terms of self-grooming time in female rats. When considering each group separately, no significant difference was found between genders. * *p* < 0.05 compared to the VPA group (within each gender separately). The triangular dots represent numerical data for the animals in each group.

The rats’ self-grooming times are presented in Figure 4. Prenatal VPA administration increased the duration of self-grooming in male rats. The self-grooming times were significantly lower in the gestation + lactation period omega-3 treatment group compared to the VPA group (*p* < 0.05). In female rats, there was no significant difference between the groups in terms of self-grooming time (*p* > 0.05). When considering each group separately, no significant difference was found between genders (*p* > 0.05).

**Figure 5:**
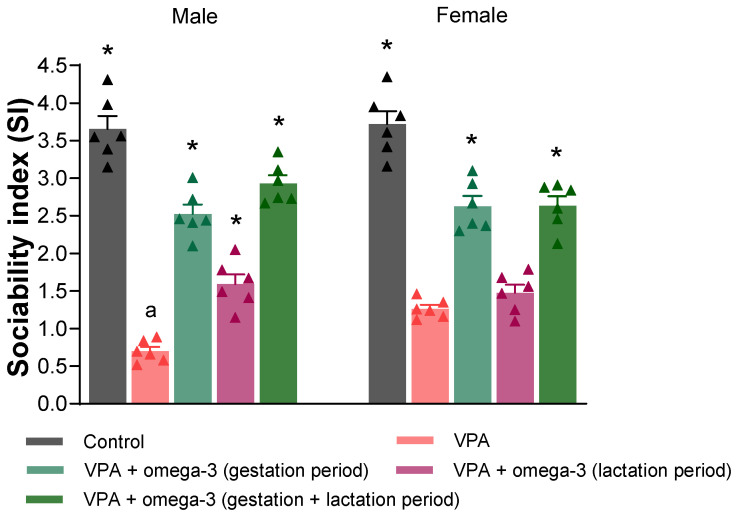
The sociability index (SI) scores of the groups. Prenatal VPA administration decreased SI scores in both males and females. In males, all treatments significantly increased SI scores. In females, the treatments except for the lactation period treatment significantly increased SI scores. Considering each group separately, the male VPA group had lower SI scores than the female VPA group. * *p* < 0.05 compared to the VPA group (within each gender separately); ^a^ *p* < 0.05 for genders (within each group separately). The triangular dots represent numerical data for the animals in each group.

**Figure 6:**
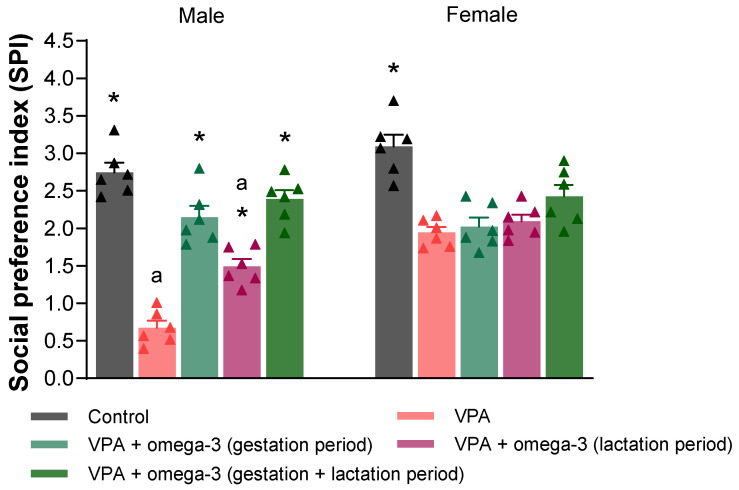
The social preference index (SPI) scores of the groups. Prenatal VPA administration decreased SPI scores in both males and females. In males, all treatments significantly increased SPI scores. However, treatments did not significantly affect SPI scores in females. Considering each group separately, the males had lower SPI scores than the females in the VPA and lactation period treatment groups. * *p* < 0.05 compared to the VPA group (within each gender separately); ^a^ *p* < 0.05 for genders (within each group separately). The triangular dots represent numerical data for the animals in each group. The triangular dots represent numerical data for the animals in each group.

Figure 5 and Figure 6 show the SI and SPI scores, respectively. In male rats, the SI and SPI scores of all groups were significantly higher than those of the VPA group (*p* < 0.05). In female rats, the SI scores of all groups except the lactation period treatment group were higher than those of VPA group (*p* < 0.05). Only the SPI scores of the control group were significantly higher than those of the VPA group (*p* < 0.05). The omega-3 treatments did not improve SPI scores in the female rats (*p* > 0.05). Considering each group separately, the male VPA group had lower SI and SPI scores than the female VPA group (*p* < 0.05). There was also a significant difference in SPI score between genders in the lactation period treatment group, to the detriment of males (*p* < 0.05).

**Figure 7:**
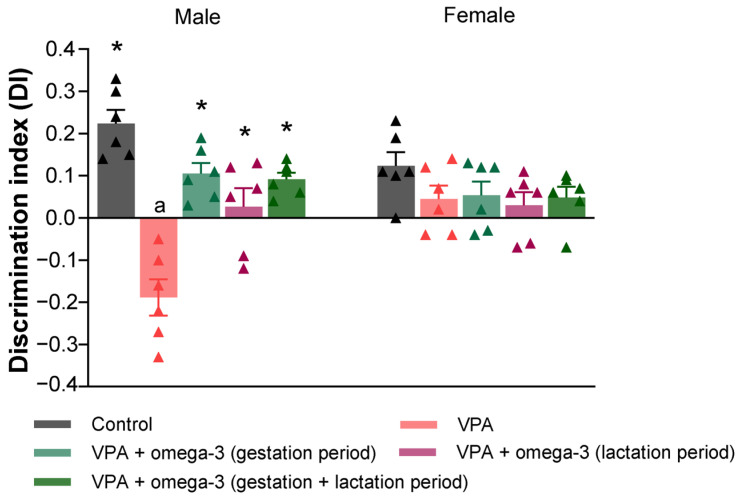
The novel object discrimination index (DI) scores of the groups. Prenatal VPA administration decreased DI scores and all treatments reversed VPA-induced DI decreases in male rats. However, no significant difference was found between the groups in terms of DI scores in female rats. Considering each group separately, the male VPA group had lower DI scores than the female VPA group. * *p* < 0.05 compared to the VPA group (within each gender separately); ^a^ *p* < 0.05 for genders (within each group separately). The triangular dots represent numerical data for the animals in each group.

The novel object discrimination index (DI) scores of the rats are shown in Figure 7. While no significant difference was found between the groups in terms of DI scores in female rats, the DI scores of all groups were significantly higher than those of the VPA group in male rats (*p* < 0.05). Moreover, considering each group separately, the male VPA group had lower DI scores than the female VPA group (*p* < 0.05).

**Figure 8:**
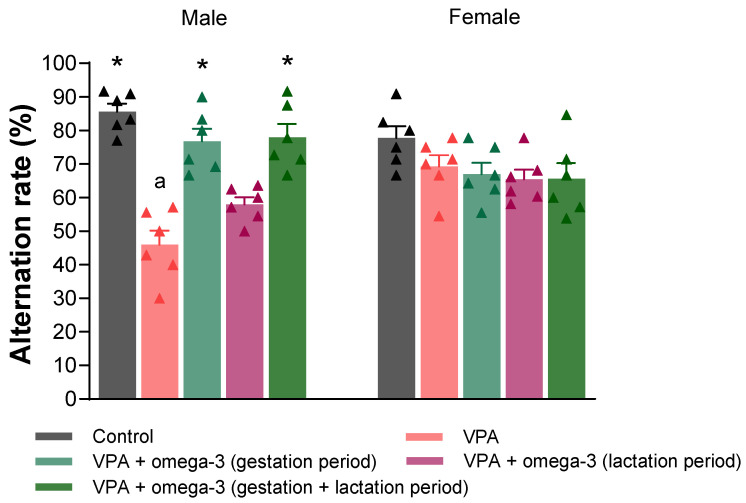
The alternation rates (%) of the groups. Prenatal VPA administration decreased alternation rates and the omega-3 treatments, except for the lactation period treatment, reversed these decreases in male rats. However, there was no significant difference between the groups in terms of alternation rates in female rats. Considering each group separately, the male VPA group had lower alternation rates than the female VPA group. * *p* < 0.05 compared to the VPA group (within each gender separately); ^a^ *p* < 0.05 for genders (within each group separately). The triangular dots represent numerical data for the animals in each group.

Figure 8 shows the alternation rates evaluated in the Y-maze test. In male rats, the alternation rates of all groups except the lactation period treatment group were significantly higher than those of the VPA group (*p* < 0.05). There was no significant difference between the groups in terms of alternation rates in female rats (*p* > 0.05). Considering each group separately, the male VPA group had lower alternation rates than the female VPA group (*p* > 0.05).

**Figure 9:**
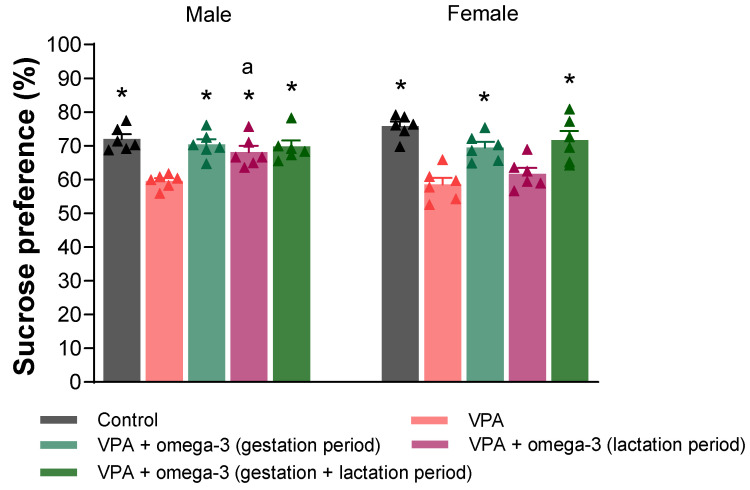
The sucrose preference rate (%) of the groups. Prenatal VPA administration decreased the sucrose preference rate in both males and females. In males, all treatments significantly increased the sucrose preference rate. On the other hand, the treatments, except lactation period treatment, increased the sucrose preference rate in females. Considering each group separately, sucrose preference rate was lower in males than in females in the lactation period treatment group. * *p* < 0.05 compared to the VPA group (within each gender separately); ^a^ *p* < 0.05 for genders (within each group separately). The triangular dots represent numerical data for the animals in each group.

The sucrose preference rate, the last behavioral parameter, is presented in Figure 9. In male rats, all groups had significantly higher sucrose preference rates than the VPA group (*p* < 0.05). In female rats, the sucrose preference rates of all groups except the lactation period treatment group were significantly higher than those of the VPA group (*p* < 0.05). Considering each group separately, sucrose preference rate was lower in males than in females in the lactation period treatment group (*p* < 0.05).

**Figure 10:**
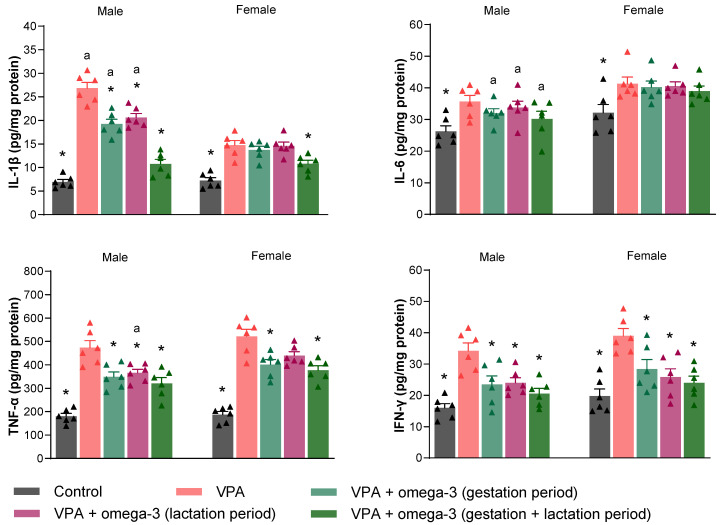
The proinflammatory mediator levels (IL-1β, IL-6, TNF-α, and IFN-γ) in the prefrontal cortex of the rats. Prenatal VPA administration significantly increased all mediator levels in the prefrontal cortex in both males and females. All omega-3 treatments decreased IL-1β, TNF-α, and IFN-γ levels in males. On the other hand, in the female rats, the gestation + lactation period treatment significantly decreased IL-1β, TNF-α, and IFN-γ levels, gestation period-only treatment significantly decreased TNF-α and IFN-γ levels, and lactation period-only treatment significantly decreased IFN-γ levels. The treatments did not significantly affect IL-6 levels in either males or females. Considering each group separately, the male VPA group had higher IL-1β levels than the female VPA group. In the gestation period and lactation period treatment groups, the males had higher levels of IL-1β and lower levels of IL-6 than the females. The males also had lower TNF-α levels than the females in the lactation period treatment group. Lastly, the IL-6 level was the only inflammatory parameter for which a difference was found in the prefrontal cortex tissues of males and females in the gestation + lactation period treatment group. * *p* < 0.05 compared to the VPA group (within each gender separately); ^a^ *p* < 0.05 for genders (within each group separately). The triangular dots represent numerical data for the animals in each group.

Figure 10 shows the proinflammatory mediator levels (IL-1β, IL-6, TNF-α, and IFN-γ) in the prefrontal cortex of the rats. Prenatal VPA administration significantly increased all proinflammatory mediator levels in the prefrontal cortex in both male and female rats (*p* < 0.05). All omega-3 treatments decreased IL-1β, TNF-α, and IFN-γ levels in male rats (*p* < 0.05). The efficacy of omega-3 treatments in female rats was slightly more limited than in male rats. The gestation + lactation period treatment significantly decreased IL-1β, TNF-α, and IFN-γ levels (*p* < 0.05), gestation period-only treatment significantly decreased TNF-α and IFN-γ levels (*p* < 0.05), and lactation period-only treatment significantly decreased IFN-γ levels (*p* < 0.05) in female rats. The treatments did not affect IL-6 levels in the prefrontal cortex in either gender (*p* > 0.05). Considering each group separately, the male VPA group had higher IL-1β levels than the female VPA group (*p* < 0.05). There was no significant difference between male and female VPA groups in terms of other proinflammatory mediators (*p* > 0.05). In the gestation period and lactation period treatment groups, the male rats had higher IL-1β levels and lower IL-6 levels than the female rats (*p* < 0.05). The male rats also had lower TNF-α levels than the female rats in the lactation period treatment group (*p* < 0.05). Finally, the IL-6 level was the only inflammatory parameter for which a difference was found in the prefrontal cortex tissues of male and female rats in the gestation + lactation period treatment group (*p* < 0.05).

**Figure 11:**
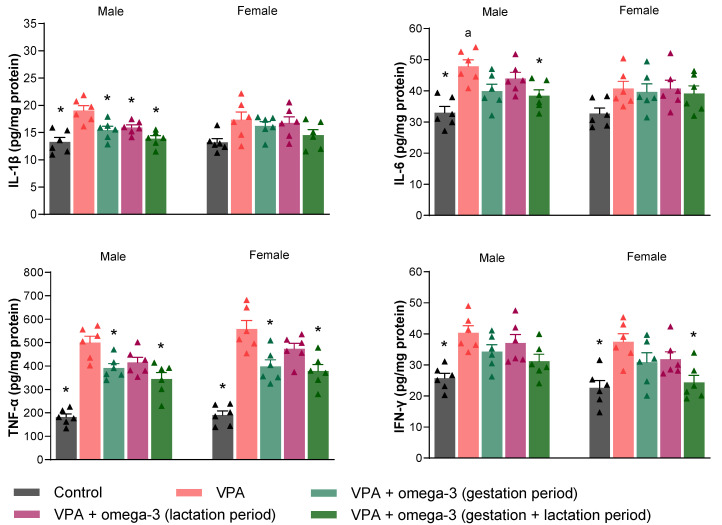
The proinflammatory mediator levels (IL-1β, IL-6, TNF-α, and IFN-γ) in the hippocampal tissues of the rats. Prenatal VPA administration significantly increased all hippocampal proinflammatory mediator levels in males. However, it did not significantly affect hippocampal IL-1β and IL-6 levels in females. All omega-3 treatments significantly decreased IL-1β levels in males. Moreover, all treatments except the lactation period treatment significantly decreased TNF-α levels in both males and females. Lastly, only the gestation + lactation period treatment significantly improved IL-6 levels in males and IFN-γ levels in females. The treatments did not affect IFN-γ levels in males or IL-1β and IL-6 levels in females. Considering each group separately, the male VPA group had higher IL-6 levels than the female VPA group. Apart from this, there was no significant difference between genders in terms of hippocampal inflammatory mediator levels in any group. * *p* < 0.05 compared to the VPA group (within each gender separately); ^a^ *p* < 0.05 for genders (within each group separately). The triangular dots represent numerical data for the animals in each group.

Proinflammatory mediator levels in the hippocampal tissues of the rats are presented in Figure 11. Prenatal VPA administration significantly increased all hippocampal proinflammatory mediator levels in male rats (*p* < 0.05). However, in female rats, only hippocampal TNF-α and IFN-γ levels increased by prenatal VPA intervention (*p* < 0.05). Whereas all omega-3 treatments significantly decreased hippocampal IL-1β levels (*p* < 0.05), gestation + lactation period treatment significantly decreased hippocampal IL-6 levels in male rats (*p* < 0.05). In contrast, the treatments did not affect hippocampal IL-1β and IL-6 levels in female rats (*p* > 0.05). The treatments except lactation period treatment significantly decreased hippocampal TNF-α levels in both male and female rats (*p* < 0.05). Lastly, gestation + lactation period treatment significantly decreased hippocampal IFN-γ levels in female rats (*p* < 0.05), whereas omega-3 treatments had no significant effect on hippocampal IFN-γ levels in male rats (*p* > 0.05). Considering each group separately, the male VPA group had higher IL-6 levels than the female VPA group (*p* < 0.05). Apart from this, there was no significant difference between genders in terms of hippocampal inflammatory mediator levels in any group (*p* > 0.05).

**Figure 12:**
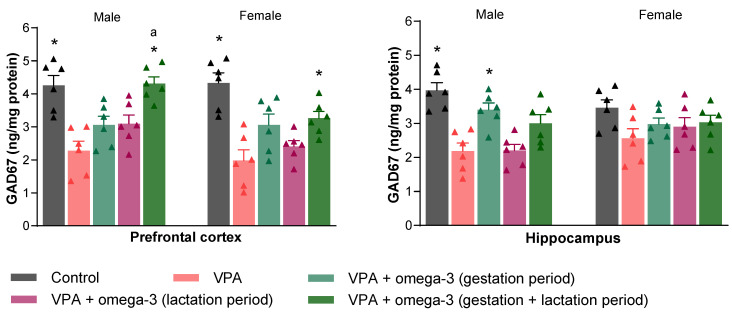
GAD67 levels in the prefrontal cortex and hippocampus of the rats. Prenatal VPA administration decreased prefrontal cortex GAD67 levels in both males and females. It also decreased hippocampal GAD67 levels in males only. The only intervention that improved prefrontal cortex GAD67 levels in both males and females was the gestation + lactation period omega-3 treatment. Only the gestation period omega-3 treatment significantly increased hippocampal GAD67 levels in males. The treatments had no significant effect on hippocampal GAD67 levels in females. Considering each group separately, the prefrontal cortex GAD67 levels of males were higher than those of females in the gestation + lactation period treatment group. * *p* < 0.05 compared to the VPA group (within each gender separately); ^a^ *p* < 0.05 for genders (within each group separately). The triangular dots represent numerical data for the animals in each group.

Figure 12 shows the GAD67 levels in the prefrontal cortex and hippocampus of the rats. Prenatal VPA intervention dramatically decreased GAD67 levels in the prefrontal cortexes of male and female rats (*p* < 0.05). It also decreased hippocampal GAD67 levels in male rats only (*p* < 0.05). The gestation + lactation period omega-3 treatment increased prefrontal cortex GAD67 levels in both genders (*p* < 0.05). Hippocampal GAD67 levels increased with the gestation period treatment in male rats (*p* < 0.05), whereas they were not significantly affected by omega-3 treatments in female rats (*p* > 0.05). Considering each group separately, the prefrontal cortex GAD67 levels of male rats were higher than those of female rats in the gestation + lactation period treatment group (*p* < 0.05). However, there was no significant difference between genders in terms of hippocampal GAD67 levels in any group (*p* > 0.05).

**Figure 13:**
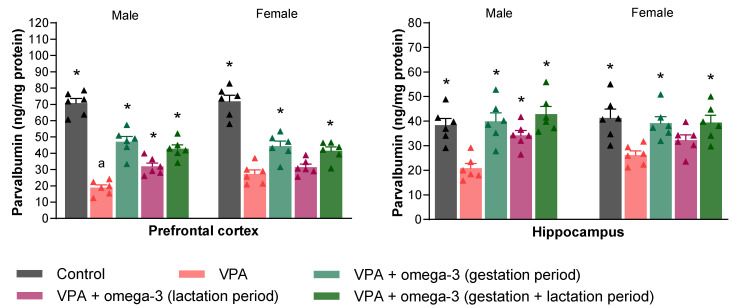
Parvalbumin levels in the prefrontal cortex and hippocampus of rats. Prenatal VPA administration decreased prefrontal cortex and hippocampus parvalbumin levels in both males and females. All omega-3 treatments significantly increased the prefrontal cortex and hippocampus parvalbumin levels in the males. All treatments except the lactation period treatment significantly increased the prefrontal cortex and hippocampus parvalbumin levels in the females. Considering each group separately, the male VPA group had significantly lower prefrontal cortex parvalbumin levels than the female VPA group. * *p* < 0.05 compared to the VPA group (within each gender separately); ^a^ *p* < 0.05 for genders (within each group separately). The triangular dots represent numerical data for the animals in each group.

The parvalbumin levels in the prefrontal cortex and hippocampus of the rats are presented in Figure 13. Prenatal VPA administration significantly decreased parvalbumin levels in the prefrontal cortex and hippocampus in both male and female rats (*p* < 0.05). In contrast, in male rats, all omega-3 treatments significantly increased parvalbumin levels in both the prefrontal cortex and hippocampus (*p* < 0.05). On the other hand, in female rats, all treatments except for the lactation period treatment significantly increased parvalbumin levels in the prefrontal cortex and hippocampus (*p* < 0.05). Considering each group separately, the male VPA group had significantly lower prefrontal cortex parvalbumin levels than the female VPA group (*p* < 0.05). However, there was no significant difference between genders in terms of hippocampal parvalbumin levels in any group (*p* > 0.05).

## 4. Discussion

The importance of preventive and therapeutic approaches to autism, the prevalence of which is increasing every year, has become clearer in recent years. The widely accepted rehabilitation methods for autism, for which there is no standard pharmacological treatment, include lifelong education, cognitive behavioral therapy, and social skills training. Complimentary treatment methods are often employed to reduce autistic behaviors and relieve some of the accompanying symptoms. It is evident that with the discovery of the important role of omega-3 fatty acids in brain health, their use in the treatment of neurological and neuropsychiatric diseases has increased. However, it has been reported that the dietary fatty acid profile present in early life is reflected in the fatty acid composition of the brain in later life [69]. This suggests that a preventative approach for early omega-3 fatty acid intake may be more effective than therapeutic omega-3 fatty acid administration.

This study focused on the possible beneficial effects of omega-3 fatty acids on autism-like behavioral and selected molecular changes induced by VPA in rats. Within the scope of this study, prenatal VPA administration impaired all behaviors in males, whereas it impaired social interaction and hedonic behavior in females. Many studies have noted that prenatal VPA exposure causes behavioral abnormalities, including those that mimic the core symptoms of autism [59,70,71,72]. However, studies involving both genders have indicated that behavioral problems are more pronounced in male animals. Male animals have been reported to exhibit more severe impairments than females in terms of parameters such as social interaction, anxiety, hypoalgesia, acoustic startle reflex, attention processing speed, and motivation as a result of prenatal VPA exposure [73,74,75]. Several mechanisms may explain why VPA does not cause behavioral changes in males and females to the same extent. The first of these is the neuroprotective effect of estrogens [76]. It is thought that estrogens, which have anti-inflammatory effects, may suppress VPA-induced neuroinflammation, especially during estrus. Another reason may be differences in corticosterone levels. It has been reported that prenatal VPA exposure increases basal corticosterone levels much more in males than in females [77]. Corticosterone can exacerbate behavioral rigidity by increasing sensitivity to stress [78]. Another important mechanism may be related to GABAergic dysfunction. In male animals, inhibitory circuits have been reported to be more severely impaired [79]. In addition, immune abnormalities, such as decreased splenic cell proliferation and thymus atrophy, have also been found to be more prevalent in males [80]. Thus, gender differences in prenatal VPA-induced behavioral symptoms may be explained by multi-layered molecular mechanisms such as hormonal regulation, GABAergic system dysfunction, and differences in immune response.

Our findings strongly suggest that the gestation + lactation period treatment has a protective effect against behavioral symptoms in males. Omega-3 treatment also showed protective effects against different behavioral symptoms when administered only during gestation or only during lactation. In females, two VPA-induced behavioral symptoms (sociability and anhedonia) improved with only gestation and gestation + lactation period treatments. Different experimental studies in the literature examining the efficacy of early omega-3 treatments have also reported improvement in behavioral symptoms associated with autism. Eshra et al. [81] reported that the consumption of an omega-3-enriched diet during gestation and lactation alleviated anxiety and social interaction disorders in a maternal high-fat diet-induced autism model. In a study using a genetic model of autism (Fmr1-Δexon 8), Schiavi et al. [82] found that consuming an omega-3-enriched diet during gestation and lactation improved social ability, assessed by the three-room sociability and social novelty test, and object memory assessed by the novel object recognition test, in male offspring. Turpin et al. [83] reported that a diet rich in EPA and DHA administered from the first day of gestation until separation attenuated VPA-induced sociability disorder and the intensity of rearing episodes in both male and female rats. Several epidemiological studies have also emphasized that early omega-3 fatty acid deficiency is associated with autistic behaviors. Steenweg-de Graaff et al. [84] reported that a reduced omega-3/omega-6 intake ratio during pregnancy was associated with autism. Another study including 258 mother–child pairs found that mothers who received higher total omega-3s in the second half of pregnancy were 40% less likely to have a child with autism [85]. None of these studies examined whether there are gender differences in the anti-autistic effects of omega-3. Our study, which presents the first findings on gender comparisons in terms of the protective effects of omega-3, shows that omega-3 treatments positively affect almost all behavioral symptoms in males but do not affect parameters other than sociability and anhedonia in females. This can be attributed to mechanisms that differ between males and females, such as neurodevelopmental trajectories, hormones, and inflammatory responses, which we mentioned above [75,76,77]. It is highly likely that the milder neural effects of prenatal VPA exposure in females mask the behavior-improving effects of omega-3. Although current findings suggest that early omega-3 treatment in males is ambitious in rescuing autism-related behavioral symptoms, further studies should focus on the impact of hormonal differences on treatment efficacy between the genders.

In recent years, a large body of evidence has accumulated pointing to an association between inflammatory mechanisms and autism. Disturbances in microglial activity during brain development have been shown to disrupt the maturation of synapses [86,87]. This has led to new hypotheses about autism [88,89]. Research on animal models strongly suggests that proinflammatory cytokines are directly associated with the pathogenesis of autism [90,91]. In this study, groups were compared in terms of IL-1β, IL-6, TNF-α, and IFN-γ concentrations in the prefrontal cortex and hippocampus. These two brain regions are critical in the pathogenesis of autism. The prefrontal cortex is involved in decision-making, language skills, and social and emotional processes. Social interaction and communication disorders, which are core symptoms of autism, are directly associated with pathology in this brain region [92,93]. The hippocampus, on the other hand, is associated with learning and spatial memory. Memory deficits, which are common in autism, are associated with this brain region [94]. In this study, prenatal VPA exposure increased IL-1β, IL-6, TNF-α, and IFN-γ levels in both the prefrontal cortex and hippocampus in males. On the other hand, hippocampal IL-1β and IL-6 levels were not significantly increased in female rats. The gestation + lactation omega-3 treatment reversed inflammation in the prefrontal cortex and hippocampus in both genders and was more effective than the gestation-only and lactation-only treatments. However, the gestation-only omega-3 treatment was also successful in alleviating neuroinflammation. The lactation-only omega-3 treatment was partially successful in males but not in females. Several studies reported that direct administration of omega-3 to offspring alleviated autism-related neuroinflammation. Jia et al. [95] found that prenatal VPA administration increased hippocampal IL-1, IL-6, and TNF-α levels in rats, whereas a fish-oil-enriched diet (omega-3/omega-6 ratio 1:5) reversed these increases. Similarly, we found in another research that 30-day omega-3 supplementation at a dose of 200 mg/kg/day reversed increased cytokine levels in the prefrontal cortex and hippocampus in prenatally VPA-exposed rats [72]. However, only Eshra et al. [81] addressed the anti-neuroinflammatory effects of early maternal omega-3 intake in an autism model. They reported that the consumption of an omega-3-enriched diet during gestation and lactation improved brain IL-6 levels in a maternal high-fat diet-induced autism model. The anti-inflammatory activity of omega-3 fatty acids has long been recognized. DHA and EPA are released from the cell membrane via phospholipase A2 (PLA2) to exert their activity. Lipoxygenase (LOX) and cyclooxygenase (COX) enzymes convert these fatty acids into bioactive compounds. These bioactive components activate the anti-inflammatory response by binding to specific receptors and altering gene expression. DHA and EPA have been reported to suppress the production of proinflammatory cytokines and improve microglial function by increasing phagocytosis, which clears debris and pathogens from the brain [96]. The studies mentioned above, which reported that omega-3 fatty acids attenuate autism-related neuroinflammation, did not examine the effect of gender. In fact, our suggestions regarding the mechanisms behind the difference between genders in terms of omega-3 treatment are in alignment with previous findings. For instance, in females, estrogens exert anti-inflammatory effects that may attenuate VPA-induced damage and potentially reduce the observable effect of omega-3 [76]. In addition, gender differences in fatty acid metabolism should not be ignored. Females have higher levels of hepatic expression of PUFA desaturase enzymes and a longer DHA half-life in plasma than males. Hence, the conversion rate of ALA to DHA is also higher in females [97]. This may provide some level of intrinsic neuroprotection in females. These biological differences may explain why prenatal VPA exposure has a more pronounced detrimental effect and why omega-3 treatment is more restorative in male rats.

Glutamate decarboxylase-67, which represents physiological GABA, is one of two isoforms of the rate-limiting GAD enzyme that converts L-glutamate to GABA and plays an important role in the regulation of GABAergic function [98]. An optimal level of this enzyme is critical for E/I balance. GAD67 deficiency in GABAergic interneurons, including parvalbumin-positive neurons, evokes overactivation of the glutamatergic system. This shifts the E/I balance toward excitation [99]. There are strong signs that the chromosome encoding the GAD67 isoform may be a susceptibility locus for autism [98]. In the present study, prenatal VPA administration decreased prefrontal cortex GAD67 levels in both genders. Hippocampal GAD67 levels also tended to decrease in both genders and were significant only in males. Recent studies also reported that prenatal VPA exposure decreased GAD67 expression in different brain regions, such as the cerebellum, hippocampus, prefrontal cortex, and temporal cortex [100,101]. Although GAD67 expression was not compared in terms of gender, it is clear that these results are consistent with our findings. The literature is not rich in findings showing a direct association between omega-3 fatty acids and GAD67. Only Wattanathorn and Thukham-Mee [102] reported that a high-dose omega-3-rich tuna oil intervention increased GAD activity in a rat model of menopause. However, there is evidence that omega-3 fatty acids may affect GABAergic neurotransmission in some brain regions [103]. It is notable that GAD67, an important component of the GABAergic system, increased in the prefrontal cortex following the gestation + lactation period omega-3 treatment. One of the mechanisms underlying the alleviation of social impairments by omega-3 fatty acids may be related to the GABAergic system. Because low GAD67 expression has been associated with social behavior deficits [104]. We suggest that omega-3 fatty acids are likely to reverse GABAergic abnormalities through anti-inflammatory mechanisms. Abnormal GABA levels are known to promote microglia to release proinflammatory cytokines [105]. On the other hand, activated microglia have been shown to displace GABAergic terminals [106]. Although it has been emphasized that chronic inflammation may be associated with E/I imbalance through GABAergic dysfunction, the mechanistic pathways involved remain mysterious [107].

Parvalbumin-positive neurons are characterized by fast spiking and projection of other neurons to their soma or axon initial segment [108]. The rapid synchronization induced by these inhibitory interneurons is responsible for the generation of high-frequency gamma oscillations, which are associated with cognitive functions such as attention, memory, and perception [21]. Functional loss of parvalbumin-positive neurons was found to cause social deficits, repetitive behaviors, and impaired motor coordination/learning in mice [109]. It has also been reported that parvalbumin-knockout (PV(−/−)) mice show developmental neuroanatomical changes associated with autism, including cortical hypertrophy and cerebellar hypoplasia [110]. Our findings revealed that prenatal VPA administration decreased parvalbumin levels in the prefrontal cortex and hippocampus in both male and female rats. This is consistent with other studies reporting that prenatal VPA administration decreased the number of parvalbumin-positive neurons in the prefrontal cortex, hippocampus, cerebellum, striatum, and amygdala [28,111,112,113,114]. Parvalbumin-positive neurons consume much more energy than other neurons to maintain their fast-spiking property. They are therefore highly sensitive to adverse, challenging conditions [115]. Even a minor metabolic stress can disrupt the synchronization of gamma oscillations driven by parvalbumin-positive neurons [116]. Hence, it is highly possible that VPA-induced inflammation attenuates parvalbumin immunoreactivity. In this case, functional loss in parvalbumin-positive neurons is inevitable. Calcium-buffering capacity decreases and action potential timing is disrupted due to parvalbumin deficiency, especially in the medial prefrontal cortex. In addition, excitatory pyramidal cells are overactivated, gamma oscillations are impaired, and synaptic noise is increased. As a result, goal-directed social cognitive processes may be affected [117,118]. The increase observed in parvalbumin levels in the prefrontal cortex and hippocampus following treatment is a valuable result. However, based on the current literature, this can only be attributed to a few mechanisms, such as omega-3′s effects on inflammation and brain-derived neurotrophic factor (BDNF). The parvalbumin-rescuing effect of omega-3 may be a consequence of its anti-inflammatory activity. Likewise, a study reported that lipopolysaccharide-induced systemic inflammation decreased hippocampal parvalbumin expression, and anti-inflammatory minocycline reversed this effect [119]. It is also possible that BDNF may mediate this positive effect of omega-3. Omega-3 supplementation has been reported to lead to a strong increase in BDNF levels. BDNF may contribute to neuronal survival and development and the functional stability of parvalbumin-positive neurons [120]. Nevertheless, further studies should be planned to decipher the molecular mechanisms underlying the improvement of parvalbumin levels by omega-3 fatty acids. Histological examination of parvalbumin-positive neurons and analysis of mitochondrial markers related to apoptosis and oxidative phosphorylation would help obtain clearer results.

The high energy demand and oxidative sensitivity of parvalbumin-positive neurons bring to mind deuterated polyunsaturated fatty acids (D-PUFAs), which are more functional than their classical form. D-PUFAs are more resistant to free radical attacks than normal fatty acids [121]. Recent studies have highlighted the neuroprotective potential of D-PUFAs [122]. They provide defense against neurodegeneration by reducing mitochondrial lipid peroxidation. Turovsky et al. [123] demonstrated that deuterated linoleic acid provides resistance to ischemia-induced cell death by stabilizing astrocytic calcium signaling and reducing oxidative damage. This finding raises the question of whether deuterated omega-3 fatty acids can provide superior protection for parvalbumin-positive neurons in autism models, particularly under inflammatory or metabolic stress conditions. The neuroprotective efficacy of deuterated omega-3 fatty acids in autism could be a promising hypothesis for future studies.

Overall, we should note that the gestation + lactation period omega-3 treatment was more effective than the gestation-only and lactation-only period treatments in rescuing prenatal VPA-induced behavioral and molecular deficits in both males and females. In addition, it is also evident that the gestation-only treatment was more successful in improving autism-like symptoms and findings than the lactation-only treatment. A systematic review examining the effects of omega-3 supplementation during gestation and lactation on neurodevelopmental processes reported that there was limited evidence of the effect of early omega-3 fatty acid intake on cognitive outcomes in children. The same report also emphasized that there was much less evidence for supplementation during lactation than during pregnancy [124]. This study is the first experimental study comparing gestation and lactation period treatments in terms of the anti-autism effects of omega-3 fatty acids. Although we found that treatment during gestation is more effective than treatment during lactation, it is even more important to identify the critical key periods of early life for the efficacy of omega-3 fatty acids and to determine the factors that affect the availability of omega-3 fatty acids for the fetus during these critical neurodevelopmental periods. Notably, there are some concerns regarding the translation of these findings into clinical practice. The adequate intake (AI) levels for omega-3 in pregnant women and lactating mothers are 1.4 and 1.3 g/day, respectively [125]. Increasing omega-3 intake to prevent autism requires caution. Factors such as metabolic changes during pregnancy and the omega-3 transport capacity of the placenta may alter the effect of omega-3 [38]. Furthermore, since very high doses of omega-3 may have systemic effects such as bleeding risk [126], predictive biomarkers or screening methods are critical for identifying high-risk pregnancies if this intervention is undertaken. Ultimately, although the results obtained in animal models are encouraging, well-designed prospective human studies that consider variables such as dosage, timing, individual differences, and safety are required.

## 5. Conclusions

This study’s findings showed that both gestational and lactational omega-3 fatty acid treatments prevented autism-related deficits to different degrees in a VPA rat model. The treatment groups were ranked as “gestation + lactation” > “gestation” > “lactation” in terms of the anti-autistic efficacy of omega-3. These beneficial effects were observed at behavioral and molecular levels in both genders. However, females were found to be slightly less responsive to omega-3 treatment. Even though no current treatment for autism is fully successful, very-low-risk complementary practices, such as omega-3 fatty acid supplementation, may provide added value to standard medical and psychological interventions to alleviate behavioral symptoms. Nevertheless, further preclinical and clinical research should focus on deciphering the critical neurodevelopmental periods in which omega-3 fatty acids are effective. Furthermore, the role of gender in the neurobiological mechanisms in which omega-3s are involved needs to be investigated.

## 6. Study Limitations

The prenatal VPA-induced autism model represents only a subset of the highly heterogeneous autism phenotype, despite being a widely used experimental model with generally accepted construct and face validity. VPA is thought to trigger autism-like behaviors through mechanisms such as epigenetic modification (e.g., histone deacetylase inhibition), oxidative stress, and impaired GABAergic transmission [26]. These mechanisms are relevant to many cases of autism but do not fully reflect the complex nature of the condition, which involves hundreds of rare and common genetic variants (e.g., *SHANK3*, *CHD8*, *NLGN3*) and a variety of environmental and immunological risk factors [127]. While the VPA model generally captures social interaction and some cognitive flexibility deficits well, it may not show other common critical features of autism, such as language impairments or atypical sensory processing [27]. Therefore, caution is required when generalizing findings from this model to the broader autism spectrum. It is also important to note that the efficacy of omega-3 fatty acids may vary depending on autism subtypes with different etiologies. For example, omega-3s, which have well-known anti-inflammatory properties, may be beneficial in cases of autism characterized by neuroinflammation and immune dysregulation [128]. However, in genetic models such as *FMR1*-knockout (fragile X syndrome), in which mGluR5 signaling and synaptic protein synthesis are dysregulated, the effects of omega-3 treatment may be different or limited [129]. Given these considerations, it would be relevant to compare genetic, immune-mediated, and environmental toxin-induced models for treatment to better understand the potential therapeutic window of omega-3s. Such approaches will help identify which subtypes of autism may respond to omega-3 interventions.

Another limitation is related to the method of omega-3 administration used in this study, in which omega-3 fatty acids were administered ad libitum via drinking water. The daily omega-3 intake of each mother rat may have varied slightly. In addition, the pups did not consume identical amounts of breast milk. These factors may have led to variability in individual omega-3 exposure. Although there was no significant difference in average omega-3 intake between groups, individual differences may have had the potential to influence the results. The omega-3 supplement was dissolved in polysorbate-20 before being added to the water. It is not known whether the use of a solvent affects the bioavailability of omega-3. It would have been relevant to examine omega-3 levels in the blood or brain to confirm the efficacy of the dosage. In addition, the inclusion of healthy groups receiving omega-3 treatment would have been noteworthy in terms of examining the independent effects of omega-3 on behavioral and neurochemical parameters. Finally, a more comprehensive examination of behavioral and neurochemical findings could have allowed for a broader assessment of therapeutic efficacy. Evaluating long-term behavioral effects in adulthood could have made the findings more insightful. Examining certain biomarkers, such as PPAR-γ, which support early brain development by playing a role in neural differentiation and synaptic plasticity, could also have been noteworthy.

## Data Availability

The raw data supporting the conclusions of this article will be made available by the authors on request.
